# Supplementary material for: Three Drug Combinations for Late-Stage Trypanosoma brucei gambiense Sleeping Sickness: A Randomized Clinical Trial in Uganda
Source: PLoS Clin Trials. 2006 Dec 8;1(8):e39. doi: 10.1371/journal.pctr.0010039 (PMC1687208; doi:10.1371/journal.pctr.0010039)
Supplement: Trial Protocol — (138 KB DOC) [file pctr.0010039.sd002.doc]

**Clinical trial comparing the therapeutic combinations [melarsoprol + nifurtimox], [melarsoprol + eflornithine] and [eflornithine + nifurtimox] in the treatment of patients suffering from *Trypanosoma brucei gambiense* human African trypanosomiasis in the meningo-encephalitic phase in Uganda**

# **Final Version: December 2000**

# **Principal Investigator: Gerardo Priotto**

Product: Melarsoprol (IV), nifurtimox (oral) and eflornithine (IV)

Version: December 2000

Design: Randomized, open, clinical equivalence trial

Study site: Omugo Treatment Center, Arua District, Uganda

Principal Investigator: Gerardo Priotto, MD, MPH

Co-Investigators: Carole Fogg, MSc; Manica Balasegaram, MD; Olema Erphas, CO; Albino Louga, LabTechnician; Francesco Checchi, MSc; Patrice Piola, MD, MPH

Data handling and analysis: Epicentre

Sponsors: Médecins Sans Frontières – French Section

Médecins Sans Frontières – International (Nobel Prize fund)

Embassy of France in Uganda

Scientific committee: Thierry Ancelle, Hôpital Cochin, Paris

Cyrus J. Bacchi, Pace University. New York City. Westchester

Philippe Buscher, Institut de Médecine Tropicale, Belgique

Marc Gastellu Etchegorry, MSF, Paris

### Pierre Cattand, WHO, Genève

Dawson Mbulamberi, NSSCP, Uganda

Christophe Paquet, Institut de Veille Sanitaire, Saint Maurice

Gaëlle Ollivier, Epicentre, Paris

Dominique Legros, Epicentre, Paris

**Summary**

**Context**

Melarsoprol is the most commonly used product for the treatment of patients suffering from human African trypanosomiasis (HAT) in the meningo-encephalitic phase. This treatment is frequently complicated by fatal reactive encephalopathy, and at the same time resistance is beginning to appear in various countries. Eflornithine is effective and better tolerated, but its availability is not guaranteed and it is difficult to use. Nifurtimox, a treatment of Chagas' disease but used in the 1980s in series of cases of HAT, is at present the only other potential candidate for the treatment of late-stage HAT.

The limited number of compounds available, the lack of prospects for the development of new products and the emergence of resistance are arguments for the use of therapeutic combinations. In addition, drug combination should reduce the dosages of the drugs used and hence improve their safety. These new protocols, however, must be validated in terms of therapeutic efficacy and clinical safety. Three therapeutic combinations are nowadays possible: [melarsoprol + nifurtimox], [melarsoprol + eflornithine] and [eflornithine + nifurtimox].

**Main objective**

To compare the following 3 therapeutic combinations in terms of therapeutic efficacy and clinical safety: A: [melarsoprol + nifurtimox], B: [melarsoprol + eflornithine] and C: [eflornithine + nifurtimox] in patients suffering from *Tbg* HAT in the meningo-encephalitic phase.

**Methods**

Randomised, open, clinical equivalence trial. The criteria of assessment will be based on the proportion of therapeutic failures in each of the groups, itself based on the presence of trypanosome(s) in the CSF, blood or lymph node fluid, and on spinal cell count. The second outcome variable will be the proportion of side effects occurring in each treatment group. The third outcome variable will be the duration of survival without signs of parasitic infestation for 24 months after treatment.

The study will be conducted in Northern Uganda.

**Study duration**

36 months

**Institutions involved**

- Ugandan National Sleeping Sickness Control Program
- Médecins sans Frontières
- Epicentre

**Introduction**

*Trypanosoma brucei gambiense* (*Tbg*) Human African Trypanosomiasis (HAT) is a major public health problem in those areas in which it is currently rife (1). This is all the more so because the political upheavals, which disrupt the operations of the health services and cause population movements encourage the reappearance of historical foci (2,3). This disease, however, is still neglected and it is estimated that the control programmes cover only 10 to 20% of the 50 million people living in endemic regions (2).

HAT, which is consistently fatal in the absence of treatment, respond to a relatively non-toxic therapeutic agent, pentamidine (Pentacarinat®), if the diagnosis is made at the hemo lymphatic phase (4). In the late stage of the disease, however, when the central nervous system is invaded by the parasite, use of arsenic derivatives such as melarsoprol (Arsobal®) remains necessary. In about 5% of cases the use of melarsoprol causes iatrogenic encephalopathy, with a fatal outcome in up to 50%.

Eflornithine (difluoromethylornithine or eflornithine, Ornidyl®), for the time being, is the only other drug registered for the treatment of late-stage HAT. The institution of treatment regimens with eflornithine, however, is difficult because of the mode of administration of the product (1 infusion of 2 hours, every 6 hours). Actually, eflornithine is no longer available on the market but the patent is under supervision of WHO (5).

Nifurtimox (Lampit®, Bayer), a product used in the treatment of Chagas disease, has only been tested empirically in HAT with contradictory results (4,6,7). This is a cheap drug, which is easy to administer (orally) but not registered for use in HAT. The type and frequency of side effects are poorly documented.

The available therapeutic spectrum for the treatment of late-stage HAT is therefore limited. Furthermore, the availability of the 3 drugs listed here is only guaranteed in the medium-term. Finally, signs of resistance to melarsoprol are beginning to appear in several areas (8,9).

The search for new approaches to the therapeutic management of late-stage HAT is therefore necessary. The limited number of compounds available, the lack of prospects for the development of new products and the emergence of resistance are arguments for the use of therapeutic combinations. In addition, the use of synergistic drug combinations in time would reduce the dosages and hence the hospitalisation period and the incidence of side effects. These new protocols, however, must be validated in terms of therapeutic efficacy and clinical safety. Three therapeutic combinations are possible today: [melarsoprol + nifurtimox], [melarsoprol + eflornithine] and [eflornithine + nifurtimox). We propose to undertake a clinical trial comparing these three combinations in terms of therapeutic efficacy and clinical safety in patients suffering from *Tbg* HAT in the meningo-encephalitic phase.

**1. Context**

**1.1. Current protocols for the treatment of HAT - Toxicity of melarsoprol.**

## Standard protocols

The treatment of HAT depends on the stage at which the disease is diagnosed.

· Pentamidine in the hemo lymphatic phase

Pentamidine isethionate, an aromatic diamine, is the reference drug used for the treatment of early-stage HAT. Patients are hospitalised and receive 4 mg/kg of pentamidine in a daily intramuscular injection for 7 to 10 days.

· Melarsoprol in the meningo-encephalitic phase

The treatment of patients with late-stage disease involves the use of melarsoprol, a trivalent arsenical derivative requiring a more intense protocol and hospitalisation for 25 days. Melarsoprol has been used almost exclusively in the treatment of late-stage HAT for more than 40 years (10). The mode of action of melarsoprol is poorly understood. According to one generally accepted theory (although recently disputed), melarsoprol acts by depriving the trypanosome of trypanothion by binding irreversibly to it. Trypanothion, which is the analogue of glutathion in trypanosomes, protects the parasite from free radicals. The pharmacokinetic of melarsoprol is poorly understood, but it appears that the half-life of the drug is 35 hours. Melarsoprol crosses the blood-brain barrier in limited quantities (10,11).

In a common protocol with Melarsoprol, patients receive three series of three intravenous injections separated by rest periods of six days. The doses of melarsoprol are progressive, from 1.8 to 3.6 mg/kg/day. Prednisolone is combined systematically, as a preventive measure against reactive encephalopathy (12).

A continuous treatment schedule (2.16 mg/kg/day for 10 days) simplifying the protocol and reducing the hospitalisation period is currently under evaluation in several countries. Preliminary results show equivalent efficacy and similar safety to the standard therapeutic regimen (13).

## Results and side effects of treatment with melarsoprol

In the late-stage disease, the cure rate after treatment with melarsoprol is estimated to be greater than 95% (10,11,14). The principal risk is that of reactive encephalopathy (5.9% of cases treated with melarsoprol in a series of 1083 patients in Zaire (15), with a high mortality rate (67.2% in the same series). In an Ivory Coast series with 350 patients (6), arsenical treatment was associated with minor side effects, such as isolated febrile episodes, pruriginous rashes and subcutaneous cellulitis, sometimes followed by necrosis at the injection site, in 11.4, 4.6 and 2.8% of cases, respectively.

MSF data from Adjumani in Northern Uganda provide the following figures: among 3110 late-stage patients treated with melarsoprol, 567 (18.2%) patients suffered side effects, including 194 (6.2%) cases of reactive encephalopathy. The lethality observed during treatment was 5.5% (170/3110). The lethality of reactive encephalopathies was 51.5% (100/194). The median duration of hospitalisation was 25 days (unpublished MSF data).

**1.2. Increase in therapeutic failures with melarsoprol**

In Arua in north-west Uganda, about 25% of cases of HAT treated for the first time with a complete course of melarsoprol result in therapeutic failures (the usual therapeutic failure rate after treatment with melarsoprol is less than 5% (10,14). In addition, repeated courses of melarsoprol in cases that had relapsed initially do not produce a cure in these patients (9,16). An identical phenomenon has appeared in Southern Sudan, in the region of Ibba, where a 6-month relapse rate of 30% has been observed (unpublished MSF data). High relapse rates after treatment with melarsoprol have already been reported in the past, particularly in Zaire (10).

Supplementary analyses on strains circulating in the Arua region have shown that the high therapeutic failure rate observed in this region was due to the appearance of melarsoprol-resistant strains of trypanosomes (17).

This phenomenon, which may extend to other sites, constitutes a major public health problem because of the lack of second-line drugs for the treatment of late-stage HAT.

**1.3. Other drugs used for the treatment of late-stage HAT**

## Eflornithine

Difluoromethylornithine (eflornithine or DFMO) has been the only new molecule introduced in the treatment of HAT over the last few decades. Eflornithine was used successfully in the treatment of late-stage HAT during the 1980s (4,10). It is a trypanostatic drug, which acts as an inhibitor of ornithine decarboxylase (an enzyme acting on the cycle of synthesis of polyamines, which themselves are involved in cell multiplication and differentiation) (10,11). This ornithine decarboxylase inhibition activity also causes a reduction in the synthesis of trypanothion, which would allow a synergistic action between eflornithine and melarsoprol. The half-life of eflornithine is short (3.3 hours) (10,11). Eflornithine readily penetrates the cerebrospinal fluid (CSF) and its concentration in the CSF is higher in adults and patients already treated with melarsoprol, probably because of the greater permeability of the blood-brain barrier (18). This might explain why a lower therapeutic failure rate is observed in patients treated with eflornithine after melarsoprol failure, but with a higher frequency of side effects (unpublished MSF data).

Eflornithine is better tolerated than melarsoprol, even if its prescription causes the occurrence of a number of side effects, all reversible and without consequences on discontinuation of treatment: pancytopenia, diarrhoea (particularly when eflornithine was taken orally), convulsions, hallucinations, insomnia, hearing loss, alopecia. In patients treated with eflornithine after melarsoprol failure, a continuous course of treatment for 7 days (100 mg/kg every 6 hours) appears to have the same efficacy as a 14-day treatment (19).

The availability of eflornithine has become erratic, the manufacturer having discontinued the production process after the recent manufacture of a final batch of product ensuring the short-term availability of the drug. However, the medium-term availability of eflornithine depends on the identification of a new laboratory prepared to take on the production.

## Nifurtimox

Although never having received marketing authorisation for the indication of the treatment of HAT, nifurtimox was used in series of cases with late-stage HAT during the 1980s, particularly in Zaire (7) and Southern Sudan (4), with contradictory results. This product, a 5-nitrofuran normally used in the treatment of Chagas' disease, is an inhibitor of the trypanothion reductase enzyme (10,11).

Nifurtimox has a short half-life, of the order of 3 hours, and must be administered orally every eight hours. In Chagas' disease, the dose of nifurtimox is 10 mg/kg/day divided in 3 doses for 30 days. Some clinicians use higher doses of 15 mg/kg/day in 3 doses for 3 months. In HAT, no treatment schedule to date has been the subject of consensus. The doses used in the past have been fairly variable:

· 120 mg per day in 3 doses for 120 days or 150 mg daily in 3 doses for 60 days in combination with suramin (in adults: i.e. more or less 6 mg/kg/day) (20).

· 4 to 5 mg/kg/day in 3 doses or 12.5 to 15 mg/kg/day in 3 doses for 60 days (18)

· 30 mg/kg/day in 3 doses for 30 days or 15 mg/kg/day in 3 doses for 60 days (7)

· 15 mg/kg/day in 3 doses for 14 to 45 days alone or in combination (20,4).

The most widely used schedule is now 15 mg/kg/day in 3 doses for 2 weeks in adults and 20 mg/kg/day in 3 doses over the same period in children under 15 years of age.

The most commonly observed side effects include anorexia and weight loss, headaches, neurological effects (central: cerebellar syndrome, convulsions, coma, confusion, insomnia, and peripheral: paresthesia, motor deficits), digestive effects (nausea, vomiting, epigastric pain) and skin rashes. The toxicity of nifurtimox appears to increase with the duration of treatment (21).

The availability of nifurtimox in the future is not guaranteed.

**1.4. Drug combinations**

The combination eflornithine + melarsoprol has proved synergistic in a murine model (22) and effective in one human case after 3 ineffective courses of treatment with melarsoprol alone and 1 ineffective course of treatment with eflornithine per os alone (23). There is a rationale for introducing melarsoprol at the end of treatment with eflornithine, as melarsoprol is more active when the trypanothion pool has been reduced by eflornithine (22).

Nitrate derivatives might potentiate the effects of arsenicals at the level of trypanothion.

In RDC, 69 patients have been treated with the combination nifurtimox + melarsoprol. One of them is dead, and no relapse was recorded after 12 months of follow-up (Philippe Buscher, personal communication)

We have no knowledge either of series of cases treated with a combination of nifurtimox + eflornithine. However, the combination of eflornithine with a nitrate derivative (free radical generator) such as nifurtimox should be beneficial in terms of the toxicity of the nitrate derivatives (reduction in dosage as a result of a synergism of action).

**2. Objectives**

## Main objective

To compare the following three therapeutic combinations in terms of therapeutic efficacy and clinical safety: A: [melarsoprol + nifurtimox], B: [melarsoprol + eflornithine] et
C: [eflornithine + nifurtimox] in patients suffering from *Tbg* HAT in the meningo-encephalitic phase.

## Secondary objective

To test IgM, a biological parameter as a predictive factor for the appearance of relapses during the follow up of the patients treated. This parameter should allow for a detection faster than traditional methods such as parasitological diagnosis.

**3. Methods**

**3.1. Study design**

Open, randomised, clinical equivalence trial

**3.2. Study population**

The study will be undertaken in the HAT treatment centre of Arua in Northern Uganda.

## Inclusion criteria

All patients suffering from *Tbg* HAT, with the following criteria:

- confirmed second-stage *T.b. gambiense* infection :
- Infection diagnosed parasitologically (blood or lymph node fluid) and white blood cells > 5/mm^3^ in cerebrospinal fluid (CSF)
- or Trypanosomes detected in the CSF [[1]](#footnote-2) with any CSF cell count
- and resident in the district
- and written consent of the patient or of one of the parents/guardians for children under 15 years of age.

The laboratory tests must have been carried out within the previous 14 days to be valid.

***Exclusion criteria***

Any patients suffering from *Tbg* HAT admitted for treatment will be excluded from the study if one of the following criteria is met:

- Confirmed first-stage *T.b. gambiense* infection :
- Infection diagnosed parasitologically (blood or lymph node fluid) and trypanosomes not detected in the cerebrospinal fluid (CSF)
- and White blood cells ≤5/mm^3^ in CSF
- Second-stage *T.b. gambiense* infection :
- Trypanosome absent from blood (or lymph node fluid) and from CSF
- Or women pregnant on inclusion
- Or previous history of HAT confirmed treated during the last 24 months
- Or impossibility of regular access to the treatment centre during the 2 years following the end of the treatment
- Or less than 10 kg of body weight
- Or refugee patient

Patients diagnosed but non included in this protocol will be treated in accordance with the Uganda National Sleeping Sickness Control Programme protocols.

## Timetable

The decision to include a patient will be taken at the time of admission (D1). Patients included in the study will be monitored during their hospitalisation and the 2 years following the end of the treatment. At the end of hospitalisation, all patients will receive a timetable with all their control appointments.

**3.3. Sample size**

## Null hypothesis

The difference in the cure rates between 2 groups obtained during the treatment of patients in the meningo-encephalitic phase is greater than 10%.

## Alternative hypothesis

The difference in the cure rates between 2 groups obtained during the treatment of patients in the meningo-encephalitic phase is less than or equal to 10%.

If the observed difference in cure rates between the 2 groups is less than 10%, the null hypothesis will be rejected and the two treatments will be considered equivalent. The risk of error will then be 5% (alpha error) because if the difference in actual cure rates between the 2 treatments is greater than 10%, there is a 95% chance of demonstrating it (alpha error). Conversely, if the observed difference in the observed cure rates between the two groups is greater than 10%, the null hypothesis will not be rejected and the 2 treatments will be considered different, at a beta error set at 20%. The allocation ratio will be uniform (1 patient in group A to 1 patient in group B to 1 patient in group C).

Assuming a 95% cure rate, a maximum difference in cure rates between the groups of 10%, an identical drop-out rate in the 3 groups, an alpha error of 5% (one-sided test) and a power of 80%, this yields a sample size of 130 patients to be included in each group, i.e. 390 patients in the study. Allowing for a 10% drop-out rate, a final figure of 145 patients per group is obtained, i.e. 435 patients in total.

**3.4. Study duration**

If a treatment centre treats about 500 late-stage patients annually, allowing for the inclusion criteria, which restrict the potential recruitment, it may be estimated that patient recruitment to the study will be completed within a maximum of 12 months. As the patients are followed up subsequently for 24 months, the total study duration is estimated at 36 months.

**3.5. Randomisation**

Patients will be randomised on D1 (inclusion date), the day on which treatment is initiated. A fixed randomisation method will be used: the probability of being assigned one or other treatment will remain constant throughout the study.

The patients’ demographic, laboratory or clinical features might change during the study because of the control programme in progress. A blocking process in the randomisation will ensure that a comparable number of patients is included in each group at all times. The blocking procedure will not be disclosed to the field team before the end of the enrolment period.

**3.6. Treatment and allocation**

Depending on their group, subjects will receive one of the treatment protocols. Patients will be treated from D1 onwards.

## Blind

The modes of administration of melarsoprol (direct IV), nifurtimox (orally) and eflornithine
(2-hour infusion) are different. A blind study is therefore not readily feasible under field conditions.

## Concomitant medications for the three groups

To improve the general fitness, all patients enrolled will be pre-treated with :

- Albendazole. To be prescribed systematically at the beginning of hospitalisation at the current protocol.
- An anti-malaria treatment with the current protocol

Metronidazole (Flagyl®) will not be used throughout hospitalisation because its side effects are the same as that of nifurtimox. If necessary, Metronidazole, Praziquantel, Ivermectine and Thiabendazole will be administered at the end of hospitalisation.

## Serum and CSF samples

A new diagnosis test is currently being developed by Antwerp Institute of Tropical Medicine. This novel tool is stage-specific and allows to follow up patients by quantitative determination of IgM in CSF. In order to collaborate in this research, samples of serum (2 tubes of 2 cc) and CSF (2 tubes of 2 cc) will be taken and then frozen immediately before and after the end of treatment. Assays of specific IgM will be performed in Europe (Antwerp Institute of Tropical Medicine).

***Melarsoprol***

Prednisolone will be co-administered systematically to patients included in groups A and B. Treatment with prednisolone will start 24 hours before the beginning of the course of melarsoprol and continued at decreasing doses of 1 mg/kg/day from D1 to D5 and 0.5 mg/kg/day from D6 to D11.

Melarsoprol will be co-administered systematically with another drug. A simplified protocol of a single series of 10 injections of melarsoprol will be used because of this combination. Patients will receive a dose of 1.8 mg/kg/day for 10 days.

## Eflornithine

Patients will receive the dosage currently used in the NSSCP/MSF Uganda programme of 100 mg/kg every 6 hours (given in a 2-hour infusion) for 7 days.

## Nifurtimox

Nifurtimox is administered orally. Dosage will be the same as that currently used in the programme for some patients, i.e.: 15 mg/kg/day for 10 days in adults and 20 mg/kg/day for 10 days in children under 15 years of age, divided in 3 doses per day.

***Dosage by treatment group***

**Table 1 : Timetable of treatment**

|  | **A** | | **B** | | **C** | |
| --- | --- | --- | --- | --- | --- | --- |
|  | **melarsoprol 1 inf / day** | **nifurtimox tabs 3/day** | **melarsoprol 1 inf / day** | **eflornithine 4 inf / day** | **eflornithine 4 inf / day** | **nifurtimox  tabs 3/day** |
| **D2** | 1.8 mg/kg | 15 mg/kg | 1.8 mg/kg | 400 mg/kg | 400 mg/kg | 15 mg/kg |
| **D3** | 1.8 mg/kg | 15 mg/kg | 1.8 mg/kg | 400 mg/kg | 400 mg/kg | 15 mg/kg |
| **D4** | 1.8 mg/kg | 15 mg/kg | 1.8 mg/kg | 400 mg/kg | 400 mg/kg | 15 mg/kg |
| **D5** | 1.8 mg/kg | 15 mg/kg | 1.8 mg/kg | 400 mg/kg | 400 mg/kg | 15 mg/kg |
| **D6** | 1.8 mg/kg | 15 mg/kg | 1.8 mg/kg | 400 mg/kg | 400 mg/kg | 15 mg/kg |
| **D7** | 1.8 mg/kg | 15 mg/kg | 1.8 mg/kg | 400 mg/kg | 400 mg/kg | 15 mg/kg |
| **D8** | 1.8 mg/kg | 15 mg/kg | 1.8 mg/kg | 400 mg/kg | 400 mg/kg | 15 mg/kg |
| **D9** | 1.8 mg/kg | 15 mg/kg | 1.8 mg/kg | - | - | 15 mg/kg |
| **D10** | 1.8 mg/kg | 15 mg/kg | 1.8 mg/kg | - | - | 15 mg/kg |
| **D11** | 1.8 mg/kg | 15 mg/kg | 1.8 mg/kg | - | - | 15 mg/kg |

## Treatment procedure

Patients included in the study will be hospitalised in the Arua HAT treatment centre throughout the treatment period.

## Side effects

In case of any side effect, the event will be recorded. The decision to withhold or to resume treatment will be left to the clinician in charge. Analysis will be done on an intention to treat basis.

The following definitions will be used for recording some of the side effects :

- Reactive encephalopathy: onset of a sudden or rapidly progressive episode of coma in a patient during treatment (Glasgow score less than or equal to 8) or status epilepticus.
- Arsobal reaction: tachycardia (pulse > 100 or 140 in children under 12 years of age), with fever > 38°C and conjunctival hyperaemia.
- Diabetes mellitus: fasting blood glucose greater than or equal to 1.3 g/l, associated with glycosuria.
- Cardiomyopathy: occurrence of an arrhythmia during treatment in a patient previously free from cardiovascular symptoms.
- Enteritis: occurrence of a diarrhoeic syndrome during treatment (three or more liquid stools per 24 hours, for three consecutive days or more) resistant to oral rehydration.

In case of occurrence of any manifestation that could lead to suspect imminent encephalopathy (severe headaches, conjunctival hyperaemia, high fever without a focus of infection or malaria parasites, isolated convulsion, behavioural disorders, etc.) the decision to withhold or to resume the treatment will be at the clinician’s discretion in accordance with the practices of the treatment centre.

## Patients withdrawal during the treatment

Patients will be withdrawn if :

- A deviation from treatments schedules is made by patients’ voluntary abandon.
- Exclusion criteria occurs at post-inclusion (see paragraph exclusion criteria in 3.2)

Patients will continue to receive the same treatment and follow-up will be done. These patients will be excluded from the analysis.

## Early stopping rules

Every hundred patients included, the three treatment groups will be compared regarding :

- the lethality,
- the proportion of therapeutic failures at the end of hospitalisation, defined by the presence of trypanosomes in the CSF and/or the blood,
- the frequency of occurrence of serious adverse reactions

If the proportion of one of these events is significantly higher in one treatment group as compared to the other groups, the recruitment in this group will be stopped.

**3.7. Follow-up of patients**

**Table 2 : Timetable of activities during the study**

| **ACTIVITIES** | **D1** | **D2-11** | **D12** | **M6** | **M12** | **M24** | **Non-scheduled days** |
| --- | --- | --- | --- | --- | --- | --- | --- |
| **Informed consent– Inclusion** | X |  |  |  |  |  |  |
| **Randomisation** | X |  |  |  |  |  |  |
| **Clinical evaluation** | X | X | X | +/- | +/- | +/- | +/- |
| **Parasitological evaluation**  Woo  QBC  GP  LP | X  X  +/-  X |  | X  +/-  X | X  +/-  X | X  +/-  X | X  +/-  X | X  +/-  X |
| **Biological evaluation**  CSF white cells counts  Haematocrit Ψ  WBC count Ψ  Differential count Ψ | X  X  X  X |  | X  X  X  X | X | X | X | X |
| **Sampling for Antwerp study**  CSF sample collection  Serum sample collection | X  X |  | X  X | X  X | X  X | X  X | X  X |
| **Treatment**  Prednisolone *  A : Melarsoprol+nifurtimox  B : Melarsoprol+eflornithine  C : eflornithine +nifurtimox | X | X  X  X  X |  |  |  |  |  |
| **New therapeutic scheme if treatment failure** |  |  | X | X | X | X | X |

Ψ Only for the first 50 subjects

* Only for treatments A or B

## Follow-up duration

Patients included in the study will be monitored for 24 months after the end of treatment.

Laboratory tests (lumbar puncture, glandular puncture, QBC, haematology) and clinical examinations will be undertaken at the end of hospitalisation and 6, 12 and 24 months after the end of the treatment.

A network of community health workers will ensure the presence of the patients included in the study at the 3 scheduled follow-up visits as far as possible. In addition, the address of each patient included in the study will be noted at the time of admission. Anyone missing a follow-up visit will be sent a reminder 3 times.

Apart from this, the patient will be allowed to come at any time in the trypanosomiasis treatment centre throughout the follow-up period.

***Lost to follow-up***

A active follow-up programme will be implemented in order to limit the proportion of lost to follow up. Any patient failing to come back for control will be contacted by a special mobile team and asked to come to the treatment centre for control.

A patient will be declared a "drop-out" if he/she misses the 24-month follow-up visit.

***Classification of responses to treatment***

**Table 2**: **Classification of responses to treatment**

| 1. **A patient will be considered « cured » at a control visit at 24 months if THE TWO following conditions are fulfilled:**   • Trypanosomes are absent from blood and CSF (and from gland juice if adenopathy)  • CSF contains <20 cells per mm^3^ |
| --- |
| 1. **A patient will be considered as « treatment failure » if at a control visit at 24 months the patient presents with ONE of the following criteria:**  - Trypanosomes are present in blood (or in gland juice if adenopathy) with any cell count in CSF - Trypanosome are present in CSF with any cell count in CSF - Cell count in CSF is ≥20 cells per mm^3^ |
| 1. **A patient will be considered as «treatment failure» at any control visit if ONE of the following conditions is fulfilled:**  - Trypanosomes are present in blood (or in gland juice if adenopathy) with any cell count in CSF - Trypanosomes are present in CSF with any cell count in CSF - Cell count in CSF is ≥20 cells per mm^3^, and has increased since the end of treatment - Cell count in CSF is ≥ 20 cells per mm^3^, and has increased two times consecutively |
| 1. **A patient will be considered « to be seen at three months » at any control visit if THE TWO following conditions are fulfilled:**  - Trypanosomes are absent from blood and CSF (and from gland juice if adenopathy) - Cell count in CSF is ≥ 20 cells per mm^3^, and has increased since the last control visit |

If a patient is not in accordance with these definitions, the final decision will be left to the clinical staff in charge.

***Decisions in the event of trypanosomiasis infection during the follow-up:***

- *For the first-stage of T.b. gambiense infection*

Patients from any groups identified positive for first-stage *T.b. gambiense* infection will be treated with pentamidine by current protocol (4 mg/kg/day during 10 days).

- *For second-stage of T.b. gambiense infection or treatment failure*

Patients from groups A and B identified as therapeutic failures will be treated with the following protocol: [eflornithine at 400 mg/kg/day for 14 days + nifurtimox at 15 mg/kg/day for 14 days]. Patients from group C identified as therapeutic failures will be treated with the following  protocol: [eflornithine with 400mg/kg/day for 14 days + melarsoprol with 2.2 mg/kg/jour for 10 days].

# **Deaths**

Causality of treatment or trypanosomiasis in the occurrence of deaths during the 2 years follow-up will be categorised as « likely », « unlikely » or « unknown ». Deaths will be analysed as follow:

- Deaths as consequence of trypanosomiasis considered « likely » will be analysed as treatment failure.
- Deaths as consequence of treatment considered « likely » will be analysed as treatment failure.
- Deaths as consequence of treatment and trypanosomiasis considered « unlikely » or « unknown » will be censored for the survival analysis and excluded from the primary end-point analysis.

**3.8. Analysis**

This will be an "intention-to-treat" analysis. The treatment groups will be compared pair-wise.

The first outcome variable is the proportion of therapeutic failures in each treatment group.

The second outcome variable is the proportion of side effects occurring in each group.

## Survival analysis

The third outcome variable will be the duration of survival without signs of parasitic infestation for 24 months after treatment. The survival curves for each group will be compared using the Kaplan Meier technique, which includes drop outs, and the log rank test.

## Multivariate analysis

Certain clinical and laboratory variables might be confounding factors, which must be taken into account during the analysis. Cox’s model (proportional hazard) will be used to obtain an estimate of the relative risk adjusted for these variables.

In an equivalence study, the point of interest is the difference observed between the two groups, assuming a 90% confidence interval (or an upper limit of 95%) around this difference (with a one-tailed test):

- If the upper limit is above 10%, the null hypothesis that there is a difference between the 2 groups cannot be rejected. If B is compared with A, it will be concluded that B is superior to A (at the 20% level).
- If the upper limit is below 0%, it will be concluded that A is superior to B (at the 20% level).
- Finally, if the upper limit is between 0 and 10%, the null hypothesis may be rejected and the alternative hypothesis accepted that the 2 groups A and B are equivalent (at the 5% level).

In the same way, for the analysis of survival, a 90% confidence interval (or an upper limit of 95%) is set around the relative hazard:

- If the upper limit is above a relative hazard of 1.12 (0.95/0.85) (i.e. 1.12 times more risk of treatment failure during the 24 months following treatment in one group than in the other), the null hypothesis that there is a difference between the 2 groups cannot be rejected.
- If the upper limit is below 1.0, it will be concluded that A is superior to B (at the 20% level).
- Finally, if the upper limit is between 1.0 and 1.12, the null hypothesis may be rejected and the alternative hypothesis accepted that the two groups A and B are equivalent (at the 5% level).

**4. Data collection**

Data will be collected by medical doctors, clinical officers and nurses involved in the programme. It will be supervised in the field by a medical doctor and by the principal investigator.

**4.1. Laboratory techniques used**

*Test for trypanosomes in the blood:*

WOO: micro-centrifugation in micro-haematocrit tubes (24)

QBC: Quantitative Buffy Coat (if WOO' s test is negative) (25)

*Test for trypanosomes in the lymph node fluid:*

Direct observation of parasites in a wet preparation

*Test for trypanosomes in the CSF:*

Double centrifugation and direct observation in micro-haematocrit tubes (26)

*Spinal cell count:*

In KOVA slides

*Haematology*

Haematocrit

WBC count, total and differential

**4.2. Data to be collected**

*Demographic data:*

Name, age, sex, address, screening method (active or passive).

*Laboratory data on inclusion:*

Result of CATT whole blood and 1/4 dilution

Results of lymph node puncture (trypanosome +/‑)

Results of parasitological examination of the blood (WOO +/‑; QBC +/‑)

Results of parasitological examination of the CSF (trypanosome +/‑)

Spinal cell count

Haematology

*Clinical data on inclusion and during hospitalisation:*

Disease history

Weight/height on admission

Weight on discharge

Temperature

Lymph nodes

Neurological signs

Intercurrent disease

Treatment received and doses

*Side effects of treatment*

*Results of treatment*

*Laboratory data on discharge:*

Results of parasitological examination of the blood (QBC +/‑)

Parasitological examination of the CSF (trypanosome +/‑)

Spinal cell count

Haematology

Specific IgM in CSF and serum (Antwerp)

*Laboratory data during follow-up:*

Results of lymph node puncture (trypanosome +/‑)

Results of parasitological examination of the blood (QBC +/‑)

Parasitological examination of the CSF (trypanosome +/‑)

Spinal cell count

Specific IgM in CSF and serum (Antwerp)

**5. Control of bias and quality control procedures**

**5.1. Bias control**

The randomisation process prevents the introduction of any bias in selection by the care teams when patients are included. However, the lack of blinding does not guarantee uniform treatment of the patients among the 3 groups.

The blocking technique used for the randomisation prevents any imbalance over time in the distribution of the number of patients between the 3 groups that might have been the source of a bias in selection. This process will only be known to the medical personnel at the end of the inclusion period.

Treatment compliance must be guaranteed by the admission of all patients to the treatment centre.

**5.2. Quality control**

A field doctor will be in charge of supervising the study procedures.

## Quality control of the clinical procedures

A written guideline will provide the medical and paramedical staff with all the necessary information for the daily running of the study (inclusion and exclusion criteria, follow-up procedures, etc.). The medical team will receive specific training and the protocol will be tested for one month in the field. The performance of the clinical procedures will be reviewed at regular intervals by the principal investigator.

## Quality control for the laboratory investigations

A code will be allocated to each patient included in the study, which will be used to identify all the laboratory results on inclusion and during follow-up.

The validity of the laboratory measurements will be evaluated periodically on samples taken in duplicate from patients drawn at random and examined blind. In addition, the laboratory measurement instruments will be calibrated regularly throughout the study.

The cell counts in the CSF will be performed twice by the same technician. When the 2 values observed differ by more than 2 cells per mm^3^, a second person will carry out a third count. The mean of the 3 observed values will be used.

## Quality control for data

The clinical and laboratory data collection questionnaires will be tested in the field before their final distribution. Data entry errors will be limited by automatic control procedures. A dual entry will be performed.

**6. Ethics, informed consent and confidentiality**

The protocol will be submitted for approval to the Uganda National Council for Science and Technology (ethics committee) and to the National Sleeping Sickness Control Program.

Each subject will be informed of the aims, methods, anticipated benefits and potential hazards of the trial. Explanations about the study (clinical trial and taking of serum and CSF samples), will be given either by the doctor or by the health assistant.

A written informed consent document, translated into the local language, will be read to each eligible patient or parent/guardian for children under 15. The patient will be asked to provide written and signed consent. Confidentiality will be guaranteed by a suitable coding system.

**7. Scientific committee**

A scientific committee will be set up prior to study initiation. The members of this committee will be entrusted with the critical appraisal of the protocol. They will be informed regularly of the progress of the study.

Members of the scientific committee:

Dr Thierry Ancelle, Hôpital Cochin, Paris

Dr Cyrus J. Bacchi, Pace University. New York City. Westchester

Dr Philippe Buscher, Institut de Médecine Tropicale, Belgique

Dr Marc Gastellu Etchegorry, MSF, Paris

### Dr Pierre Cattand, WHO, Genève

Dr Dawson Mbulamberi, National Sleeping Sickness Control Program, Uganda

Dr Christophe Paquet, Institut de Veille Sanitaire, Saint Maurice

Dr Gaëlle Ollivier, Epicentre, Paris

Dr Dominique Legros, Epicentre, Paris

**8. Administrative references**

**8.1. Institutions involved**

- National Sleeping Sickness Control Program of Uganda
- Médecins sans Frontières, 8 rue Saint Sabin, 75 011 Paris
  Telephone: 00 33 1 40 21 29 29, Fax: 00 33 1 48 06 68 68

Po Box 2362, Kampala, Uganda, Telephone: 256 41 267 881

- Epicentre, 8 rue Saint Sabin, 75 011 Paris
  Telephone: 00 33 1 40 21 28 48, Fax: 00 33 1 40 21 28 03
  Po Box 2362, Kampala, Uganda, Telephone/Fax: 256 41 269 998

**References**

1. Penchenier L, Sarda J, Jannin J. [Where is the focus of human trypanosomiasis in Mossaka (Congo)]. Bull Soc Pathol Exot 1993;86(5):347-50.
2. Kuzoe FA. Current situation of African trypanosomiasis. Acta Trop 1993;54(3-4):163-8.
3. Mbulamberi DB. Possible causes leading to an epidemic outbreak of sleeping sickness: facts and hypotheses. Ann Soc Belg Med Trop 1989;69(Suppl 1):217-220.
4. Van Nieuwenhove S. Advances in sleeping sickness therapy. Ann Soc Belg Med Trop 1992;72(Suppl 1):7-12.
5. O.M.S. Nouveau médicament contre la maladie du sommeil. Communiqué O.M.S./64, 1990.
6. Doua F, Yapo FB. Human trypanosomiasis in the Ivory Coast: therapy and problems. Exp Parasitol 1993;77(3):306-14.
7. Pepin J, Milord F, Meurice F, Ethier L, Loko L, Mpia B. High-dose nifurtimox for arseno-resistant Trypanosoma brucei gambiense sleeping sickness: an open trial in central Zaire. Res Vet Sci 1992;52(3):292-8.
8. Legros D, Evans S, Maiso F, Enyaru JC, Mbulamberi D. Risk factors for treatment failure after melarsoprol for Trypanosoma brucei gambiense trypanosomiasis in Uganda. Trans R Soc Trop Med Hyg 1999;93(4):439-42.
9. Legros D, Fournier C, Gastellu Etchegorry M, Maiso F, Szumilin E. [Therapeutic failure of melarsoprol among patients treated for late stage T.b. gambiense human African trypanosomiasis in Uganda]. Bull Soc Pathol Exot 1999;92(3):171-2.
10. Pepin J, Milord F. The treatment of human African trypanosomiasis. Adv Parasitol 1994;33:1-47.
11. Richard E. Thérapeutique de la trypanosomiase africaine : revue des médicaments anciens, présents et en cours d''expérimentation. . Paris: Université René Descartes. Faculté de médecine de Cochin Port-Royal., 1999. 303 p.
12. Pepin J, Milord F, Guern C, Mpia B, Ethier L, Mansinsa D. Trial of prednisolone for prevention of melarsoprol-induced encephalopathy in gambiense sleeping sickness [see comments]. Lancet 1989;1(8649):1246-50.
13. Burri C, Nkunku S, Merolle A, Smith T, Blum J, Brun R. Efficacy of new, concise schedule for melarsoprol in treatment of sleeping sickness caused by Trypanosoma brucei gambiense: a randomised trial. Lancet 2000 Apr 22;355(9213):1419-25.
14. Pepin J, Milord F, Khonde A, Niyonsenga T, Loko L, Mpia B. Gambiense trypanosomiasis: frequency of, and risk factors for, failure of melarsoprol therapy. Cent Afr J Med 1994;40(7):186-92.
15. Pepin J, Milord F, Khonde AN, et al. Risk factors for encephalopathy and mortality during melarsoprol treatment of Trypanosoma brucei gambiense sleeping sickness. Bull Soc Pathol Exot 1995;88(1):38-41.
16. Legros D, Enyaru J, Evans S, Maiso F, Paquet C. An outbreak of relapses among patients treated for Trypanosoma brucei gambiense trypanosomiasis in Arua, Northern Uganda. 24th ISCTRC meeting. Maputo - Mozambique, 1997.
17. Legros D, Fournier C, Kaminsky R, et al. Therapeutic failure of melarsoprol among patients treated for late-stage T.b. gambiense Human African Trypanosomiasis in Uganda. 25th ISCTRC meeting. Mombasa, Kenya, 1999.
18. Moens F, De Wilde M, Ngato K. [Clinical trial of nifurtimox in human African trypanosomiasis]. Med Trop (Mars) 1984;44(2):149-54.
19. Khonde N, Pepin J, Mpia B. A seven days course of eflornithine for relapsing Trypanosoma brucei gambiense sleeping sickness. Trans R Soc Trop Med Hyg 1997;91(2):212-3.
20. Milord F, Loko L, Ethier L, Mpia B, Pepin J. Eflornithine concentrations in serum and cerebrospinal fluid of 63 patients treated for Trypanosoma brucei gambiense sleeping sickness. Trans R Soc Trop Med Hyg 1993;87(4):473-7.
21. Janssens PG, De Muynck A. Clinical trials with "nifurtimox" in African trypanosomiasis. Ann Soc Belg Med Trop 1977;57(4-5):475-80.
22. Jennings FW. Chemotherapy of trypanosomiasis: the potentiation of melarsoprol by concurrent difluoromethylornithine (eflornithine) treatment. Trans R Soc Trop Med Hyg 1988;82(4):572-3.
23. Simarro PP, Asumu PN. Gambian trypanosomiasis and synergism between melarsoprol and eflornithine: first case report. Trans R Soc Trop Med Hyg 1996;90(3):315.
24. Woo PT. The haematocrit centrifuge technique for the diagnosis of African trypanosomiasis. Trans R Soc Trop Med Hyg 1970;64(4):523-30.
25. Bailey JW, Smith DH. The use of the acridine orange QBC technique in the diagnosis of African trypanosomiasis. Trans R Soc Trop Med Hyg 1992;86(6):630.
26. Cattand P, Miezan BT, de Raadt P. Human African trypanosomiasis: use of double centrifugation of cerebrospinal fluid to detect trypanosomes. Bull World Health Organ 1988;66(1):83-6.

1. If lumbar puncture is traumatic (with blood flux), it must be done again. [↑](#footnote-ref-2)
