# Supplementary material for: Three Drug Combinations for Late-Stage Trypanosoma brucei gambiense Sleeping Sickness: A Randomized Clinical Trial in Uganda
Source: PLoS Clin Trials. 2006 Dec 8;1(8):e39. doi: 10.1371/journal.pctr.0010039 (PMC1687208; doi:10.1371/journal.pctr.0010039)
Supplement: Alternative Language Abstract S1 — (28 KB DOC) [file pctr.0010039.sd003.doc]

**Spanish**

**Tres Combinaciones Terapéuticas para la Enfermedad del Sueño por *Trypanosoma brucei Gambiense* en fase tardía: Ensayo Clínico Randomizado en Uganda**

# Resumen

**Objetivos:** Comparar la eficacia y seguridad de tres combinaciones de drogas para el tratamiento de la tripanosomiasis humana africana por *Trypanosoma brucei Gambiense* en fase tardía.

**Diseño:** ensayo clínico randomizado, abierto, con control activo, paralelo, comparando tres brazos.

**Sitio:** Centro de Tratamiento de la Enfermedad Del Sueño manejado por Médicos Sin Fronteras en Omugo, Distrito de Arúa, Uganda

**Participantes:** Pacientes en estadio 2 diagnosticados en el norte de Uganda

**Intervenciones:** Melarsoprol-nifurtimox, melarsoprol-eflornitina y nifurtimox-eflornitina. Las dosis eran uniformes: melarsoprol IV 1.8 mg/kg/día, diariamente por 10 días; eflornitina IV 400 mg/kg/dia, cada 6 horas por 7 días; nifurtimox oral 15 o 20 (niños <15 años) mg/kg/día, cada 8 horas por 10 días. Los pacientes fueron seguidos por 24 meses.

**Resultados a medir:** Tasa de curación y reacciones adversas atribuíbles al tratamiento.

**Resultados:** 54 pacientes fueron randomizados antes de suspender el enrolamiento debido a toxicidad inacceptable en uno de los tres brazos. Las tasas de curación obtenidas en el análisis por intención de tratar fueron de 44.4%, 78.9% y 94.1% respectivamente, y significativamente mayores con nifurtimox-eflornitina (p=0.003) y melarsoprol-eflornitina (p=0.045) comparadas con melarsoprol-nifurtimox. Las reacciones adversas fueron menos frecuentes y menos severas con nifurtimox-eflornitina, resultando en menos interrupciones del tratamiento y menor mortalidad. Hubo cuatro muertes con melarsoprol-nifurtimox y una con melarsoprol-eflornitina.

**Conclusiones:** La combinacion nifurtimox-eflornitina se destaca como una prometedora terapia de primera línea que puede hacer progresar la terapéutica de la enfermedad del sueño, a pesar de que este estudio interrumpido no permite sustentar interpretaciones concluyentes. Estudios más extensos son necesarios para continuar su evaluación.
