# Supplementary material for: Three Drug Combinations for Late-Stage Trypanosoma brucei gambiense Sleeping Sickness: A Randomized Clinical Trial in Uganda
Source: PLoS Clin Trials. 2006 Dec 8;1(8):e39. doi: 10.1371/journal.pctr.0010039 (PMC1687208; doi:10.1371/journal.pctr.0010039)
Supplement: Alternative Language Abstract S2 — (29 KB DOC) [file pctr.0010039.sd004.doc]

**French**

##### Trois Combinaisons Thérapeutiques pour la

**Maladie du Sommeil à *Trypanosoma brucei Gambiense***

**en phase tardive: Essai Clinique Randomisé en Ouganda**

# Résumé

**Objectifs:** Comparer l’efficacité et la sécurité de trois combinaisons de médicaments pour le traitement de la trypanosomose humaine africaine à *Trypanosoma brucei Gambiense* en phase tardive.

**Design:** essai clinique randomisé, ouvert, avec contrôle actif, parallèle, comparant trois bras.

**Lieu:** Centre de Traitement de la Maladie Du Sommeil géré par Médecins Sans Frontières à Omugo, District d’Arua, Ouganda

**Participants:** Patients en stade 2 diagnostiqués en Nord-Ouganda

**Interventions:** Mélarsoprol-nifurtimox, mélarsoprol-éflornithine et nifurtimox-éflornithine. Les dosages étaient uniformes: mélarsoprol IV 1.8 mg/kg/jour, quotidien pendant 10 jours; éflornithine IV 400 mg/kg/jour, toutes les 6 heures pendant 7 jours; nifurtimox per os 15 ou 20 (enfants <15 ans) mg/kg/jour, toutes les 8 heures pendant 10 jours. Les patients ont été suivis pour 24 mois.

**Résultats mesurés:** Taux de guérison et événements indésirables attribuables au traitement.

**Résultats:** 54 patients ont été randomisés avant de suspendre les inclusions en raison de la toxicité inacceptable dans l’un des trois bras. Les taux de guérison obtenues avec l’analyse en intention de traiter étaient de 44.4%, 78.9% et 94.1% respectivement, et étaient significativement supérieurs avec nifurtimox-éflornithine (p=0.003) et mélarsoprol-éflornithine (p=0.045) comparés avec mélarsoprol-nifurtimox. Les événements indésirables étaient moins fréquents et moins sévères avec nifurtimox-éflornithine, résultant en moins d’interruptions du traitement et en une moindre mortalité. Il y a eu quatre décès avec mélarsoprol-nifurtimox et un avec mélarsoprol-éflornithine.

**Conclusions:** La combinaison nifurtimox-éflornithine se révèle comme une thérapie de première intention prometteuse pouvant apporter un progrès dans la thérapeutique de la maladie du sommeil, même si cette étude interrompue ne permet pas de sustenter des interprétations concluantes. Des études incluant plus de sujets sont nécessaires afin de continuer son évaluation.
