# Supplementary material for: Three Drug Combinations for Late-Stage Trypanosoma brucei gambiense Sleeping Sickness: A Randomized Clinical Trial in Uganda
Source: PLoS Clin Trials. 2006 Dec 8;1(8):e39. doi: 10.1371/journal.pctr.0010039 (PMC1687208; doi:10.1371/journal.pctr.0010039)
Supplement: Alternative Language Abstract S3 — (22 KB DOC) [file pctr.0010039.sd005.doc]

**Portuguese**

Três combinações terapeûticas para a doença do sono por *Trypanosoma brucei Gambiense* em segunda fase:

um ensaio clínico randomizado em Uganda

### Resumo

**Objectivos**: comparar a eficácia e a segurança de três combinações de medicamentos para o tratamento da segunda fase da trypanossomíase humana africana causada por *Trypanosoma brucei Gambiense*.

**Desenho do estudo**: ensaio clínico randomizado, aberto, de controlo activo, paralelo, comparando três braços.

**Sítio**: Centro de Tratamento da Doença do Sono dirigido por Médicos Sem Fronteiras em Omugo, distrito de Arua, Uganda.

**Participantes**: doentes em fase 2 diagnosticados no norte de Uganda.

**Intervenções**: melarsoprol-nifurtimox, melarsoprol-eflornitina e nifurtimox-eflornitina. Dosagens foram uniformes: IV melarsoprol 1.8 mg/kg/dia, uma vez por dia durante 10 dias; IV eflornithine 400 mg/kg/dia, de 6 em 6 horas durante 7 dias; nifurtimox oral 15 ou 20 (crianças <15 anos) mg/kg/dia, de 8 em 8 horas durante 10 dias. Os doentes foram fiscalizados durante 24 mêses.

**Resultados medidos**: taxa de cura e eventos adversos devidos ao tratamento.

**Resultados**: 54 doentes foram randomizados antes de parar o recrutamento por causa de toxicidade inaceitável num dos 3 braços. Taxas de cura obtidas através da analise por intenção-de-tratar foram 44.4%, 78.9% and 94.1% respectivamente e foram significativamente superiores com nifurtimox-eflornitina (p=0.003) e melarsoprol-eflornitina (p=0.045) comparados com melarsoprol-nifurtimox. Os eventos adversos foram menos frequentes e menos severos com nifurtimox-eflornitina resultando em menos interrupções do tratamento e falecimentos. Houve quatro mortes com melarsoprol-nifurtimox e uma com melarsoprol-eflornitina.

**Conclusões**: a combinação nifurtimox-eflornitina revela-se como uma terapia de primeira intenção prometedora que podería trazer um progresso no tratamento da doença do sono, apesar deste ensaio interrupto não permitir interpretações conclusivas. Necessitam-se de estudos utilizando amostras populacionais maiores para continuar a sua avaliação.
